# Supplementary material for: Mechanism of Action of Ketogenic Diet Treatment: Impact of Decanoic Acid and Beta—Hydroxybutyrate on Sirtuins and Energy Metabolism in Hippocampal Murine Neurons
Source: Nutrients. 2020 Aug 8;12(8):2379. doi: 10.3390/nu12082379 (PMC7468807; doi:10.3390/nu12082379)
Supplement: Supplementary file 1 [file nutrients-12-02379-s001.pdf]

Mechanism of Action of Ketogenic Diet Treatment: Impact of Decanoic Acid and Beta – Hydroxybutyrate on Sirtuins and Energy Metabolism in Hippocampal Murine Neurons

Partha Dabke, Anibh Das

Supplementary material – Table S1 (list of primers)

**Primer**	**Sequence (5′ – 3′)**
SIRT1 (forward)	*CAG ACC CTC AAG CCA TGT TTT*
SIRT1 (reverse)	*GAT CCT TTG GAT TCC TGC AA*
SIRT2 (forward)	*TTG CCA AGG AGC TCT ATC CC*
SIRT2 (reverse)	*GTG TGA TGT GTA GAA GGT GCC*
SIRT3 (forward)	*ACT TCC GCT AAA CTT CTC CCG*
SIRT3 (reverse)	*CCC ACA CAG AGG GAT ATG GG*
SIRT4 (forward)	*TCG ATA GCT CCG CAT TAT GTC AA*
SIRT4 (reverse)	*GCG GCA CAA ATA ACC CCG AG*
MCTr 1 (forward)	*TTG GGT TCT GTG TCT ACG CC*
MCTr 1 (reverse)	*GAG GCG GCC TAA AAG TGG T*
MCTr 2 (forward)	*CTC CTC TGG CTG GTA AAT TGC*
MCTr 2 (reverse)	*GGC CTC CAT TTC TCT GGA CG*
ACTB (forward)	*ACT CTG TGT GCA TCG GTG GC*
ACTB (reverse)	*CAG CTC AGT AAC AGT CCG CC*
HPRT1 (forward)	*GCT TAC CTC ACT GCT TTC CG*
HPRT1 (reverse)	*CAT CAT CGC TAA TCA CGA CGC*
B2M (forward)	*TGT ATG CTA TCC AGA AAA CCC C*
B2M (reverse)	*GCA TTT CAA TGT GAG GCG GG*
